# Need to have more Biomedical Journals with Impact Factor and importance of Publication Audit

**DOI:** 10.12669/pjms.35.4.1300

**Published:** 2019

**Authors:** Shaukat Ali Jawaid

For the fourth consecutive year Pakistan Journal of Medical Sciences has retained the No. 1 position of having the highest Impact Factor among the biomedical journals published from Pakistan. In fact it has further improved and the current Impact Factor is 0.834 and the number of citations has also increased as compared to the previous year as per the Journal Citation Report released by the Clarivate Analytics from USA. The accompanying Table-I also gives the details of Impact Factor of other journals from Pakistan covered by Clarivate Analytics and the number of citations.^1^ We are extremely grateful to the members of the Editorial Board, International Advisors as well as our valued reviewers because we could achieve all this due to their devotion and dedication and it would not have been possible without their continued patronage, help, guidance and assistance which they have been extending in honorary capacity.

**Table I:** Journals from Pakistan with Impact Factor (2018)

Full	Journal Title	Total Cites	Journal Impact Factor	Eigen factor Score
1	PAKISTAN VETERINARY JOURNAL	1,123	1.36	0.00127
**2**	**PAKISTAN JOURNAL OF MEDICAL SCIENCES**	**2,095**	**0.834**	**0.00368**
3	INTERNATIONAL JOURNAL OF AGRICULTURE AND BIOLOGY	2,793	0.802	0.00211
4	PAKISTAN JOURNAL OF ZOOLOGY	1,621	0.79	0.00198
5	INTERNATIONAL JOURNAL OF PHARMACOLOGY	840	0.754	0.00078
6	PAKISTAN JOURNAL OF BOTANY	4,376	0.672	0.00264
7	JOURNAL OF THE PAKISTAN MEDICAL ASSOCIATION	3,344	0.642	0.00317
8	PAKISTAN JOURNAL OF AGRICULTURAL SCIENCES	869	0.618	0.00113
9	PAKISTAN JOURNAL OF PHARMACEUTICAL SCIENCES	2,021	0.596	0.00258
10	JOURNAL OF ANIMAL AND PLANT SCIENCES	1,620	0.529	0.00239
11	JCPSP-JOURNAL OF THE COLLEGE OF	1,948	0.407	0.00187
	PHYSICIANS AND SURGEONS PAKISTAN			
12	JOURNAL OF THE CHEMICAL SOCIETY OF PAKISTAN	896	0.393	0.00086

Impact Factor we have always believed is just one and not the only one criterion to judge the quality and standard of a medical journal^.2-3^ We in Pakistan have about a dozen high quality peer reviewed biomedical journals but they could not get IF because till a few years ago most of these journals were being managed by non-professionals who did not know the importance of such indexations like IF. Now these journals are being edited by professionals, have applied and they hopefully will get the IF in the coming few years. It is taking time because the Clarivate Analytics has also made it a bit more difficult as now the new journals are first covered in eSCIE (emerging Science Citation Index) for a few years, their publication and performance is monitored before they are shifted to SCIE and start getting Impact Factor and it can take from three to five years.

Even otherwise IF is being given too much importance by the regulatory authorities all over the world and Pakistan is no exception. However, if regulatory authorities overseas are helping and encouraging the local journals to get indexed in important databases and earn IF, we do not see any such effort in Pakistan on the part of our regulatory agencies. Iran is just one example. Ever since the formation of Medical Journals Commission which has some professional editors as its members and is located at the Ministry of Health, the number of journals indexed in various databases and those enjoying Impact Factor has increased simply because they were helped and guided by the Commission. In Pakistan, we have two important regulatory bodies i.e. Pakistan Medical & Dental Council (PM&DC) and the Higher Education Commission (HEC). They do want more journals to enjoy Impact Factor and the HEC in particular has made it mandatory for the faculty members in universities as well as those doing PhD to publish their research work in Impact Factor Journals but do not help journals acquire IF which can only be done through professional capacity building of the editors. After the reconstitution of the PM&DC Journals Committee for which credit goes to Prof. Arshad Javed Vice Chancellor of Khyber Medical University and the former acting President of PM&DC Mr. Justice Shakirullah Jan that it did start some training workshops for Editors. At least two such workshops were held at Khyber Medical University Peshawar and King Edward Medical University Lahore but then the new PM&DC Council was nominated after the promulgation of the PM&DC Ordinance 2018 and this training programme could not be continued for various reasons. The HEC also made some-half-hearted efforts to organize training for the Editors, exposing them to the working of Open Journal System (OJS) and the use of OJS by journals has also been made mandatory but the overall performance of both these regulatory bodies’ remains sub-optimal. We have often highlighted as to what they are supposed to do to help the biomedical journals instead of just providing some financial grants as done by the HEC but without much success.^2^ We do need more biomedical journals with Impact Factor to help authors and relieve pressure on the three medical journals which enjoy Impact Factor. At present it has created a serious situation not only for the authors but also for the editors of IF journals that have to work under tremendous pressure, stress and strains because they cannot entertain beyond a particular number of manuscripts in view of their human resource as well as financial constraints. This naturally annoys a large number of authors whose manuscripts are not accepted for further processing while getting manuscript published in Impact Factor Journals overseas is very expensive which many authors from low income countries including Pakistan cannot afford.^4^-^7^

## PUBLICATION AUDIT

We do Publication audit of Pakistan Journal of Medical Sciences annually. We believe it is a useful tool to evaluate progress of the journal and also plan for the future.^8^ A critical analysis for the year 2018 will reveal that Pakistan Journal of Medical Sciences attracted 1719 submissions from numerous countries. Table-II. Details of cities from where we received papers for publication during the same period from Pakistan are shown in Table-III. Out of this three hundred eleven papers (311) were published, 1211 were not accepted for further processing due to various reasons, four were rejected because of plagiarism, seventeen papers were withdrawn by the authors as they were impatient to get it published immediately and we could not accommodate them. In some cases if the topic of the study was interesting, the write up was good and did add some useful information to the medical literature but the similarity index score was a bit high after checking with iThenticate, they were asked to revise it, reduce the similarity index score to the acceptable limits before the paper was accepted for further processing as we try to practice an author friendly policy. Table-IV.

**Table II:** Country wise submissions in PJMS during 2018

Country	Total
Algeria	1
Argentine	1
Aruba	1
Australia	2
Bahamas	2
Bangladesh	1
China	308
Cyprus	5
Ecuador	1
Egypt	12
Ghana	1
India	14
Indonesia	26
Iran	73
Iraq	15
Italy	1
Jordan	1
Kazakhstan	1
Korea	15
Malaysia	6
Nigeria	5
**Pakistan**	**436**
Palestine	1
Romania	1
Saudi Arabia	98
Serbia	1
South Africa	2
Sudan	1
Taiwan	1
Thailand	3
Turkey	657
Turkmenistan	1
United Arab Emirates	5
United Kingdom	7
United States	5
Viet Nam	4
Yemen	1
Hungary	1
Italy	1
Belgium	1

**Grand Total**	**1719**

**Table III:** City wise submissions from Pakistan during 2018 in PJMS

City	Total
Abbottabad	1
Azad Kashmir	4
Bahawalpur	12
Bannu	1
Chitral	1
Dera Ghazi Khan	1
Dera Ismail Khan	2
Faisalabad	17
Gilgit	1
Gujranwala	3
Gujrat	1
Haripur	3
Hyderabad/ Jamshoro	8
Islamabad	37
Karachi	157
Kohat	5
Lahore	94
Larkana	2
Lodhran	1
Mansehra	1
Mardan	2
Mohmand District	1
Multan	4
Muzafarabad	2
Nawabshah	1
Peshawar	40
Quetta	4
Rahim Yar Khan	2
Rawalpindi	20
Saidu, KP	1
Sargodha	1
Sialkot	3
Swat	1
Wah Cantt.	2

**Grand Total**	**436**

**Table IV:** PJMS manuscripts statistics of 2018 at a Glance.

Published Articles:	311 (18%)
Rejected Articles:	1211(70.5%)
Rejected article on similarity report:	4(0.2%)
Withdraw Articles:	17(1%)
Under Process:	176(10.3%)
Received Articles:	1719(100%)

As usual highest number of submissions during 2018 was received from Turkey (657) as against 508 during 2017. Submissions from Pakistan during 2018 were (436) as against (453) during the last year which shows a decrease simply because we continue to raise the standard bar every year as the number of submissions increases. Submissions from China during 2018 were (308) as against (331) during 2017. Submissions from Iran were one hundred three which increased from 73 in 2017. We have seen a progressive increase in the number of submissions from Saudi Arabia 98 in 2018 from 62 in 2017. Table-II.

Submissions from within the country were highest from Karachi 157 followed by Lahore 94, Peshawar 40, Islamabad 37, Rawalpindi 20, Faisalabad 17 and Bahawalpur 12. Table-III. This also reflects more academic activity and interest in research, medical writing in these major cities as per submissions that we have received. In fact a large number of submissions from most of the cities are accommodated by other biomedical journals which are not so stringent with the peer review system and quality control, as such it is not possible to have exact status of interest in research and Publications among the healthcare professionals affiliated with various medical institutions all over the country.

The number of papers published from Pakistan during 2018 was 311 and as expected most of them were original articles (266) followed by Case Reports (13), Short Communications (6) Editorials, Review articles and Viewpoints four each. Most of the issues published during 2018 had between 49 to 54 manuscripts. Table-V. If we look at country wise distribution of manuscripts published during 2018, highest number were from Pakistan (150) followed by Turkey (72), China (43) and Saudi Arabia (24) Table-VI. Overall we have made further progress in attracting quality manuscripts not only from Pakistan but also from overseas. However, still there are numerous hurdles to cross and trying to maintain and further improve the standard is not going to be an easy task particularly in view of the tremendous increase in cost of production due to devaluation of the Pakistani currency and appreciation in the value of US dollar as we have to pay in foreign exchange for most of the services that we have acquired from overseas i.e. iThenticate for plagiarism check, CrossRef and CrossCheck for Digital Object Identified (DOI), preparation of XML files for submission to PubMed Central to be visible on the PubMed. Constant efforts will be needed to overcome some of these problems for which we expect the help and assistance of the members of the Editorial Board, our valued Reviewers and some understanding on the part of authors. If authors are a bit careful, read and follow instructions for authors on the website of the journal to which they wish to submit their manuscript, it will not only minimize trauma to their manuscripts but will also make the life of the editors a bit comfortable.^9^

**Table V:** Category Wise Manuscript Published in 2018 in PJMS

Category	Jan-Feb 2018	Mar-Apr 2018	May-Jun 2018	Jul-Aug 2018	Sep-Oct 2018	Nov-Dec 2018	Total
Original Articles	44	46	41	43	45	47	266
Case Reports	1	3	3	3	1	2	13
Short Communication			2	1	1	2	6
Editorial	1			1	1	1	4
Review Article		2	1			1	4
View Point	1		1		1	1	4
Conference Proceedings		1		1	1		3
Correspondence			1	1	1		3
Leading Article			1		1		2
Systematic Review			1	1			2
Clinical Case Series				1			1
Guest Editorial	1						1
Workshop Proceedings				1			1
Retraction	1						1

**Grand Total**	**49**	**52**	**51**	**53**	**52**	**54**	**311**

**Table VI:** Country Wise Manuscript Published in 2018 in PJMS

Category	Jan-Feb 2018	Mar-Apr 2018	May-Jun 2018	Jul-Aug 2018	Sep-Oct 2018	Nov-Dec 2018	Total
Pakistan	26	28	20	21	25	30	150
Turkey	10	14	17	11	7	13	72
China	10	3	6	8	11	5	43
Saudi Arabia	2	4	4	7	5	2	24
Iran				2		1	3
Cyprus		1		1	1		3
Iraq					1	1	2
Korea				2			2
United Arab Emirates		2					2
Thailand	1					1	2
Malaysia				1			1
Bangladesh						1	1
India					1		1
United Kingdom			1				1
Romania			1				1
Australia			1				1
Serbia and Montenegro			1				1
UAE					1		1

**Grand Total**	**49**	**52**	**51**	**53**	**52**	**54**	**311**

The authors also need to clear some of their misconceptions. It is a well-known fact that “Gift Authorship” is very common in many countries including Pakistan and every effort needs to be made to check this menace. PM&DC has now decided to give equal credit to six authors in a manuscript but this does not mean that every manuscript can have six authors. There is a clearly laid down criteria for authorship by International Committee of Medical Journal Editors (ICMJE) and most good quality peer review journals follow this with little bit of modification.^10^ Hence, those submitting the manuscripts have to be very careful as regards authorship and enlist only those as authors who are eligible for authorship otherwise they risk their manuscripts being rejected from most reputed peer reviewed biomedical journals.^11^

During 2018, we received seventy eighty requests from authors for fast track processing of their manuscripts, a facility which is available in a very few selected cases i.e. if the main author has to appear in CPSP Fellowship Examination or defense of PhD Thesis. Only fifteen such genuine requests were entertained. Some authors believe that by opting for fast track processing perhaps their manuscript will be published early which is not at all true. The sooner these authors clear their misconception, the better it will be for them. In fact it is the authors and the quality of the manuscript which decides the time frame of its further processing and publications as every editor is keen to publish good quality manuscripts which also have better chances of further citations as it will help the journal to improve its Impact Factor.
